# THE POTENTIAL CLINICAL AND ECONOMIC IMPACTS OF OVER-THE-COUNTER HEARING AIDS IN THE UNITED STATES

**DOI:** 10.1093/geroni/igac059.3123

**Published:** 2022-12-20

**Authors:** Ethan Borre, Mohini Johri, Judy Dubno, Juliessa Pavon, Evan Myers, Howard Francis, Osondu Ogbuoji, Gillian Sanders Schmidler

**Affiliations:** Duke University, Durham, North Carolina, United States; Duke University, Durham, North Carolina, United States; Medical University of South Carolina, Charleston, South Carolina, United States; Duke University School of Medicine, Durham, North Carolina, United States; Duke University, Durham, North Carolina, United States; Duke University, Durham, North Carolina, United States; Duke University, Durham, North Carolina, United States; Duke University, Durham, North Carolina, United States

## Abstract

Food and Drug Administration regulations (released 8/16/22) for over-the-counter (OTC) hearing aids (HAs) allow the purchase of OTC HAs for adults with perceived mild-to-moderate hearing loss. Given this new development, the quality-of-life benefits and costs of OTC devices are now relevant but also unknown. We sought to project the population-level costs and effectiveness of US OTC HA provision. We used a microsimulation of hearing loss (DeciBHAL-US) to simulate: 1) traditional HA provision validated to published estimates of HA uptake (10-year delay), and 2) OTC HA provision for persons with perceived mild-to-moderate hearing loss at increased uptake rates (5-year delay) across quality-of-life and cost combinations. Simulated adults experienced probabilities of hearing loss (0.1–10%/year), subsequent age- and severity-dependent HA uptake (traditional HAs: 0.5–8%/year; OTC HAs: 1–16%/year) and discontinuation (4–13%/year). Utility benefit for traditional HAs was +0.11, and was +0.05–0.11 for OTC HAs. Costs included traditional HA uptake ($3,690; maintenance=$1,000/year) and OTC HA uptake ($600-$2,000; maintenance=$220–500/year). Our model projected lifetime discounted quality-adjusted-life-years (QALYs) for traditional HAs of 18.140/person, and 18.139–18.174/person for OTC HAs varying with utility benefit (+0.05–0.11). As long as OTC HA utility benefit was >+0.05, increased access resulted in higher QALYs and was cost-effective ($4,000/QALY-$92,500/QALY, varying with HA cost and utility benefit). Our results were sensitive to variations in OTC HA uptake rates. In conclusion, OTC HAs may increase population health and access to hearing healthcare in a cost-effective manner, and our model may continue to explore the costs and benefits of OTC HAs as pricing and evidence evolves.

